# Prevention of omalizumab for seasonal allergic rhinoconjunctivitis: a retrospective cohort study

**DOI:** 10.3389/fimmu.2022.913424

**Published:** 2022-07-28

**Authors:** Rui Tang, Shubin Lei, Liping Zhu, Yuzhen Lv, Hong Li

**Affiliations:** ^1^ Department of Allergy, Peking Union Medical College Hospital, Chinese Academy of Medical Sciences & Peking Union Medical College, Beijing Key Laboratory of Precision Medicine for Diagnosis and Treatment of Allergic Disease, National Clinical Research Center for Dermatologic and Immunologic Diseases(NCRC-DID), Beijing, China; ^2^ Eight-year program of Clinical Medicine, Chinese Academy of Medical Sciences & Peking Union Medical College, Beijing, China; ^3^ Department of Allergy, Peking Union Medical College Hospital, Chinese Academy of Medical Sciences and Peking Union Medical College, Beijing, China; ^4^ Pneumology Department, Yangquan Coal Industry (Group) General Hospital, Shanxi, China

**Keywords:** omalizumab, seasonal allergic rhinoconjunctivitis, preventative injection, allergic rhinitis, allergic conjunctivitis

## Abstract

**Background:**

Allergic rhinoconjunctivitis (ARC) is an allergic disease that is characterized by conjunctival and nasal symptoms such as edema and congestion of conjunctiva, rhinorrhea, sneezing, and blocked nose. Seasonal ARC (SARC) is usually induced by seasonal allergens and often occurs at specific times during the year. Traditional treatments of SARC include nasal corticosteroids, antihistamines, and mast cell membrane stabilizers. Biological agents such as omalizumab have also been proved effective in the treatment of SARC.

**Objectives:**

We aim to certify the preventative efficacy of omalizumab for SARC and explore its influence factors.

**Methods:**

Medical records of 64 SARC patients were retrospectively analyzed, and generalized linear models were used to analyze influence factors of efficacy of omalizumab.

**Results:**

Compared with forepassed pollen season without omalizumab treatment, the combined symptom and medication score (CSMS) of ARC with pre-seasonal omalizumab was significantly lower (with omalizumab: 0.67[0.00,1.83], without omalizumab: 4.00[2.83,4.96], p<0.001, max score=6). Subgroup analysis was conducted to explore the influence factor of preventative efficacy of omalizumab. The CSMS with omalizumab treatment were not significantly different among different age, gender, dosage, number of injections, and injection date subgroups (p>0.05).

**Conclusion:**

Pre-seasonal omalizumab treatment could significantly relieve SARC related symptoms and reduce medication use. This preventative efficacy would not be influenced by the dosage and number of injections of omalizumab. A single dose of 150mg omalizumab could achieve a satisfactory outcome.

## Introduction

Allergic rhinoconjunctivitis (ARC) is a kind of allergic disease caused by a hypersensitivity reaction mediated by IgE. The conjunctival symptoms of ARC include edema and congestion of conjunctiva, watery secreta, and swelling eyelid, and the nasal symptoms of ARC include rhinorrhea, sneezing, and blocked nose (1). These symptoms could impact patients’ life quality and work efficiency (2), aggravating the disease burden. For teenage patients, ARC-related symptoms could also affect study efficiency and school performance (3). Seasonal allergic rhinoconjunctivitis (SARC) is usually caused by seasonal allergens. Previous studies showed that in America, more than 16% of the population would be affected by SARC each year, approximately 70% of which is induced by various kinds of pollen (4), therefore the prevalence of SARC is higher during the pollen season in spring and autumn. Considering the influence of SARC to patients’ life quality, control and prevention of SARC related symptoms is the emphasis of disease management. Common symptom-controlling medication includes nasal corticosteroids, antihistamine, and mast cell membrane stabilizer (5, 6). Moreover, more researches have been focusing on regarding allergen immunotherapy as a novel method of treatment and prevention, the allergen could be administrated sublingually or subcutaneously. It has been confirmed the immunotherapy is beneficial for the relief of symptoms and the reduction of drug consumption (7). In recent years, biological agents have been widely utilized, of which omalizumab is a typical representative. As a specific IgE antibody, omalizumab has been used in the treatment of allergic asthma and urticaria. It has been certified that omalizumab has the advantage of high security and less adverse effect in the treatment of allergic disease, and it could be effective for patients who do not respond to traditional antiallergic treatment. In Japan, omalizumab has been approved for the treatment of severe pollinosis (8). Relevant studies have found that omalizumab could significantly relieve the symptoms of pollinosis patients (9). Our research aims to analyze the prevention function of administration of omalizumab before pollen season and its effect factors.

## Method

### Cohort and self-contrast method

A total of 73 outpatients of SARC in Peking Union Medical College Hospital who received preventative omalizumab treatment before the start of autumn pollen season (9^th^, August, 2021) in 2021 have been included. Telephone follow-up was available for 64 of them to access the control of SARC-related symptoms. A control group was recruited from 32 SARC patients who received traditional pharmacotherapy instead of omalizumab with matched age and gender distribution, to compare the efficacy between pre-seasonal omalizumab and traditional pharmacotherapy. The diagnosis of ARC was according to the *Chinese guidelines for diagnosis and treatment of allergic rhinitis (2022, revisio*n), patients should meet the following three criteria to have a confirmed diagnosis of ARC: 1)symptoms: have ≥ 2 symptoms: paroxysmal sneezing, watery nasal mucus, itchy nose and blocked nose lasting more than 1 hour in a day, which could be complicated with lacrimation, itchy eyes, and red eyes; 2)physical sighs: paleness or swelling of nasal mucosa, watery nasal secreta; 3) allergen test: positive for at least 1 kind of pollen in skin puncture and/or positive result for serologic specific IgE test (10). Specific IgE levels were detected by enzyme-linked immunosorbent assays (ImmunoCAP system). Patients who received regular omalizumab for the treatment of other allergic diseases before the autumn pollen season in 2021 were excluded from this research.

### Dosage and administration of omalizumab

All patients received subcutaneously administrated omalizumab treatment (150mg, 300mg, 450mg, 600mg per injection;1-4 injections), the dosage was determined by clinical physicians mainly according to serum IgE level. The timing of injections was determined according to doctors’ preference.

### Assessment of efficacy

The combined symptom and medication score (CSMS) was utilized to access the efficacy of omalizumab, which is composed of a symptom score and a medication score. The symptom score is based on nasal symptoms including itchy nose, sneezing, runny nose, and blocked nose, and conjunctival symptoms including itchy/red eyes and watery eyes (1: mild symptoms, 2: moderate symptoms, 3: severe symptoms, max score of 3, i.e.18 points/divided by 6 symptoms). The medication score is based on oral and/or topical (eyes or nose) nonsedative H1antihistamines (H1A), intranasal corticosteroids (INS) with/without H1A, and oral corticosteroids with/without INS, with/without H1A, with a max score of 3 (11). For patients who are complicated with allergic asthma, an asthma control test (ACT) was conducted to evaluate the preventative efficacy of omalizumab for asthma symptoms (12).

### Ethics

This research was approved by the ethics committee of the Chinese Academy of Medical Sciences, Peking Union Medical College Hospital (No. S-k2046). Omalizumab was considered and administrated only after informed consent was conducted orally by allergy specialists to assure that all patients completely agree with the treatment of omalizumab.

### Statistical analysis

The normality of continuous data was tested by the Shapiro-Wilk test. Normal variables were presented as (average ± standard deviation [SD]) and analyzed by t-test. Nonnormal variables were presented as (median [first quantile, third quantile]) and analyzed by nonparametric tests. Generalized linear models were used to analyze influence factors of efficacy of omalizumab. P-value was considered statistically significant when<0.05. Data were analyzed using SPSS26.0.

## Results

A total of 64 SARC patients who received at least one course of preventative omalizumab treatment before the autumn pollen season were included (**Table 1**). Telephone follow-ups have been conducted to evaluate the control of SARC-related symptoms. According to the time of the first injection, all patients have been divided into three subgroups, and there were no significant differences in gender, age, or baseline CSMS among the three subgroups.

**Table 1:** Baseline information of participants .

	Omalizumab treatment					Traditional pharmacotherapy	P-value
	2 weeks in advance (n=24)	2-4weeks in advance (n=20)	4-10weeks in advance (n=20)	In total (n=64)	p-value		
Age	33.5 ± 2.7	34.9 ± 3.4	32.3 ± 3.8	33.5 ± 1.9	0.849	31.9 ± 2.8	0.589
Female (%)	11 (45.8%)	14 (70.0%)	9 (45.0%)	34 (53.1%)	0.189	16(50.0%)	0.830
Male (%)	13 (54.2%)	6 (30.0%)	11 (55.5%)	30 (46.9%)		16(50.0%)
Baseline CSMS	3.67[2.67,4.79]	4.50[3.04,5.00]	4.17[2.83,5.00]	4.00[2.83,4.96]	0.099	2.67[2.00,3.63]	0.000
Injection times							
1	8 (33.3%)	6 (30.0%)	4 (20.0%)	18 (28.1%)			
2	15 (62.5%)	9 (45.0%)	8 (40.0%)	32 (50.0%)			
3	1 (4.2%)	5 (25.0%)	7 (35.0%)	13 (20.3%)			
4	0	0	1 (5.0%)	1 (1.6%)			

**Figure 1:**
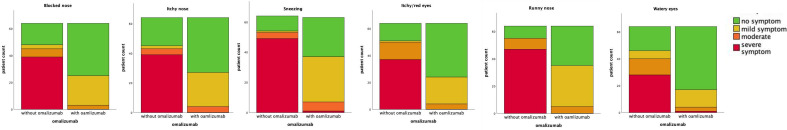
Preventative omalizumab treatment could improve clinical symptom of SARC patients significantly.

By comparing the SARC-related symptom with and without omalizumab treatment, we found that preventative injection of omalizumab could significantly relieve the symptom and reduce medication consumption (**Table 2**; **Figure 1**).

**Table 2:** Preventative omalizumab treatment could improve clinical symptoms of SARC patients significantly.

	Without preventative omalizumab treatment	With preventative omalizumab treatment before autumn pollen season	p-value
(Total) daily symptom score (dSS)	2.67[1.00,3.00]	0.33[0.00,0.83]	0.000
CSMS	4.00[2.83,4.96]	0.67[0.00,1.83]	0.000

dSS, daily symptom score.

CSMS, combined symptom and medication score.

We compared the efficacy of controlling SARC-related symptoms between pre-seasonal omalizumab and traditional pharmacotherapy. Our results show that patients who received pre-seasonal omalizumab have more severe baseline manifestation but lower CSMS after treatment (**Table 3**).

**Table 3:** Pre-seasonal omalizumab is more effective than traditional pharmacotherapy.

	Pre-seasonal omalizumab	Traditional pharmacotherapy	P-value
Baseline CSMS	4.00[2.83,4.96]	2.88[2.25,4.25]	0.000
CSMS after treatment	0.67[0.00,1.83]	1.42[0.71,2.29]	0.017
CSMS improvement^1^	2.75 ± 1.42	1.22 ± 0.99	0.000

^1^CSMS improvement: CSMS after treatment – baseline CSMS.

Furthermore, 37 patients who received omalizumab were complicated with allergic asthma, of which 4 did not have any related symptoms and did not use any asthma-controlling drug (ACT score=25) when the research was conducted. For the other 33 patients, all reported a remission of allergic asthma after preventative treatment of omalizumab, except for one patient whose ACT score was equal to that of pollen season without omalizumab treatment, the difference was statisticly significant (without omalizumab: 20.0[14.5,22.6], with omalizumab: 25.0[24.0,25.0], p<0.001). Patients with ACT scores between 20-24 were defined as well-controlled. The rate of well-controlled patients was 54.5% without omalizumab and increased to 100% after preventative omalizumab.

In order to explore the impact of gender, age, date of first injection, number of injections, dosage of omalizumab, complicated allergy, and serous total IgE(tIgE) level on the efficacy of omalizumab, participants were divided into several subgroups according to these parameters. Statistic results indicated that all these factors had no influence on the efficacy of preventative omalizumab treatment (**Table 4**).

Table 4Influence factor analysis about efficacy of preventative omalizumab.

**Table 4A:** Influence of demographic factors.

	tIgE(KU/L)	CSMS without omalizumab	CSMS with omalizumab	CSMS improvement	Recovery rate^1^
**Gender**					
Female(n=34)	158.0[69.4,349.0]	4.42[3.29,5.00]	0.59[0.00,2.00]	3.01 ± 0.24	10(29.4%)
Male(n=30)	144.0[117.0,504.8]	3.17[2.75,4.92]	1.00[0.00,1.67]	2.46 ± 0.26	9(33.3%)
P-value	0.538	0.040	0.754	0.120	0.959
**Age^2^**					
6-18 years old (n=13)	225.0[115.5,576.5]	3.67[2.75,5.00]	1.00[0.00,1.67]	2.61 ± 1.42	4(36.4%)
≥18 years old (n=51)	132.0[78.1,277.3]	4.09[2.96,4.87]	0.67[0.00,1.87]	2.78 ± 1.43	15(29.4%)
P-value	0.324	0.762	0.872	0.705	1.000

**Table 4B:** Influence of dosage, timing and injection times of omalizumab.

	tIgE(KU/L)	CSMS without omalizumab	CSMS with omalizumab	CSMS improvement	Recovery rate^1^
**Date of first injection**					
2 weeks in advance (n=24)	129.0[74.9,216.0]	3.67[2.67,4.79]	0.50[0.00,1.67]	2.69 ± 0.25	7(29.2%)
2-4 weeks in advance (n=20)	234.5[70.3,643.5]	4.50[3.04,5.00]	0.83[0.00,2.34]	2.78 ± 0.36	6(30.0%)
4-10 weeks in advance (n=20)	160.0[114.0,513.8]	4.33[2.83,5.00]	1.00[0.09,1.92]	2.79 ± 0.33	6(30.0%)
P-value	0.468	0.235	0.729	0.923	0.998
**Injection times**					
Single time (n=18)	205.0[108.9,337.5]	4.00[2.96,4.54]	0.75[0.00,2.00]	2.42 ± 1.18	5(27.8%)
Multiple times (n=46)	129.0[74.9,417.0]	4.42[2.79,5.00]	0.59[0.00,1.67]	2.88 ± 1.49	14(30.4%)
P-value	0.585	0.513	0.458	0.243	0.834
**Dosage^3^**					
150mg(n-14)	63.0[40.3,100.0]	4.83[2.83,5.00]	1.00[0.33,2.00]	2.57 ± 0.50	2(14.3%)
300mg(n=39)	153.0[114.5,484.5]	3.17[2.42,4.25]	0.67[0.00,1.67]	2.49 ± 0.25	13(33.3%)
450-600mg(n=11)	389.5[239.3,1372.0]	3.84[3.50,4.63]	0.34[0.00,0.96]	3.44 ± 0.47	4(36.4%)
P-value	0.000	0.253	0.442	0.634	0.354

**Table 4C:** Influence of complicated allergy.

	tIgE(KU/L)	CSMS without omalizumab	CSMS with omalizumab	CSMS improvement	Recovery rate^1^
**Mould allergy**					
Without mould allergy (n=31)	162.0[104.1,448.0]	2.67[1.00,3.00]	1.00[0.17,2.00]	2.54 ± 1.27	7(22.6%)
Complicated with mould allergy (n=7)	153.0[119.0,183.0]	2.67[2.00,2.83]	0.83[0.33,1.83]	2.69 ± 1.22	1(14.3%)
P-value	0.907	0.825	0.854	0.789	1.000
**Dust mite allergy**					
Without dust mite allergy (n=34)	158.0[99.1,398.5]	2.67[1.00,3.00]	0.92[0.13,1.87]	2.69 ± 1.44	8(23.5%)
Complicated with dust mite allergy (n=5)	197.0[136.5,1065.5]	0.92[0.83,2.50]	0.17[0.00,0.50]	3.67 ± 1.10	2(40.0%)
P-value	0.218	0.581	0.334	0.255	0.587
**Number of autumn pollen allergen**					
Single allergen (n=14)	115.5[58.4,274.5]	3.00[2.83,4.21]	0.33[0.00,2.00]	2.63 ± 0.29	5(35.7%)
Multiple allergen (n=50)	162.0[104.0,435.0]	4.50[3.00,5.00]	0.83[0.00,1.83]	2.79 ± 0.22	14(28.0%)
P-value	0.138	0.095	0.323	0.676	1.000

**Table 4D:** Influence of tIgE before omalizumab treatment.

	CSMS without omalizumab	CSMS with omalizumab	CSMS improvement	Recovery rate^1^
**tIgE**				
≤100KU/L(n=16)	4.00[2.87,4.96]	0.33[0.04,2.50]	2.69 ± 0.40	4(25.0%)
>100KU/L(n=45)	4.00[2.83,4.92]	0.67[0.00,1.75]	2.77 ± 0.21	14(31.1%)
P-value	0.805	0.684	0.839	0.757

1: recovery: CSMS score=0 after preventative omalizumab treatment.

2:6-18 years old: 12.0 ± 1.0 years old; ≥18: 39.0 ± 1.6 years old.

3: Patients’ weight was not significantly different among 3 dosage subgroups.

CSMS: combined symptom and medication score.

CSMS improvement: CSMS without omalizumab-CSMS with omalizumab.

In order to analyze the influence of tIgE and eosinophils (eos) to the efficacy of preventative omalizumab treatment, univariate analysis was conducted and the result showed that both tIgE and eos had no impact on CSMS improvement (p>0.05).

## Discussion

By retrospective analysis of medical records of 64 SARC patients, we certified the preventative efficacy of pre-seasonal injection of omalizumab. For patients complicated with allergic asthma, omalizumab is also efficient for the relief of asthma-related symptoms. Currently, there is no research focusing on the influence factor of the preventative use of omalizumab. We conducted subgroup analysis and univariant analysis to explore this question. Based on the statistical results, we first propose that all factors above have no influence on the preventative efficacy of omalizumab.

As a specific antibody of IgE, omalizumab has been approved for the treatment of chronic urticaria and moderate-to-severe allergic asthma. In 2001, Casale et al. reported the preventative function of pre-seasonal omalizumab followed by during-seasonal maintaining treatment for pollinosis (13). In 2020, omalizumab was approved for the treatment of cedar pollinosis (8). The latest studies have shown that in terms of relieving symptoms and improving life quality, preventative administration of omalizumab is better than the routine strategy (14). Furthermore, a previous study indicated that omalizumab could improve the control of asthma for SARC patients complicated with allergic asthma (15, 16), which is consistent with our result.

We compared the efficacy between pre-seasonal omalizumab and traditional pharmacotherapy, the result showed that in our center, pre-seasonal omalizumab has been used more frequently in patients with more severe symptoms. In addition, pre-seasonal omalizumab was more effective in controlling SARC-related symptoms, which is consistent with previous research (14).

We innovatively explored the influence factor for the preventative efficacy of omalizumab. No significant factor was found. However, it is notable that although the baseline symptoms of female patients seem to be more severe, there is no significant difference between the response of pre-seasonal omalizumab in male and female participants.

Currently, the research focusing on the injection date is largely insufficient, relevant studies prefer to give the first omalizumab injection 1-2 weeks before the pollen season. Based on the pharmacokinetics study, serum concentrations peak 1 week after administration, and the serum elimination half-life is approximately 17-26 days (17). Therefore, it seems reasonable to give the first injection 1-2 weeks before the start of omalizumab pollen season. However, there was no significant difference in the efficacy of the three subgroups (2 weeks, 2-4weeks, and 4-10weeks in advance). More studies would be needed to determine the best time for the first omalizumab injection. Besides, we found that the dosage and number of injections have no influence on efficacy either. According to our findings, one dose of omalizumab is sufficient to achieve a satisfactory outcome. In conclusion, 150 mg in the 2 weeks before the start of pollen season is efficient for the prevention of SARC-related symptoms, which could not only reduce the financial burden but also avoid time and energy consumption of multiple visits to the hospital.

Relevant research found that for the treatment of spontaneous urticaria and persistent asthma, patients with higher tIgE and eos levels had a better response to omalizumab (18, 19). However, in our study, tIgE and eos level have no influence on the preventative efficacy of omalizumab. This may be explained by the fact that, compared with baseline level, the change of tIgE and eos after omalizumab injection had a more predictable value for treatment response.

It is still an open question whether the number of allergen sensitizations has an effect on the efficacy of omalizumab in treating allergic disease. As for asthma, some scholars believe that the number of allergen sensitizations would not affect the outcome of omalizumab treatment (20), while other researchers reported that patients who are sensitized to ≥4 different groups of aeroallergens have a higher exacerbation rate (19). In our research, we found that for SARC, the number of allergen sensitizations and the complicated allergy of dust mite or mold would not impact the preventative efficacy of omalizumab.

In conclusion, this study certified omalizumab as a pre-seasonal preventative treatment for SARC and pointed out that the date, number of injections, and dosage of omalizumab would not influence the efficacy of omalizumab, suggesting that a single injection of 150mg omalizuab within 2 weeks before pollen season would be effective enough for the prevention of SARC-related symptoms. Our work could reduce the disease burden for SARC patients. Moreover, we proposed that the precautionary effect would not be impacted by the level of tIgE, eos, and the type and number of allergen sensitizations, which is inconsistent with previous research. Our work indicates that as a preventative treatment, omalizumab could be widely used for most moderate-to-severe SARC patients.

## Data availability statement

The original contributions presented in the study are included in the article/supplementary material. Further inquiries can be directed to the corresponding author.

## Ethics statement

This research was approved by the Ethics Committee of Peking Union Medical College Hospital, Chinese Academy of Medical Sciences & Peking Union Medical College. The approval number of the Ethics Committee is S-k2046. Because the patients did not receive any clinical intervention, written informed consent was not required for this research.

## Author contributions

HL participated in the design of the study and provided guidance in manuscript writing. RT participated in the design of the study, discussion of the result, writing the manuscript and revision of the article. SL participated in the gather of original data, statistic analysis, writing of the manuscript and revision of the article. LZ and YL participated in the follow-up of patients and statistic analysis. All authors contributed to the article and approved the submitted version.

## Conflict of interest

The authors declare that the research was conducted in the absence of any commercial or financial relationships that could be construed as a potential conflict of interest.

## Publisher’s note

All claims expressed in this article are solely those of the authors and do not necessarily represent those of their affiliated organizations, or those of the publisher, the editors and the reviewers. Any product that may be evaluated in this article, or claim that may be made by its manufacturer, is not guaranteed or endorsed by the publisher.
